# Protocol for a mobile laboratory study of co-administration of cannabis concentrates with a standard alcohol dose in humans

**DOI:** 10.1371/journal.pone.0277123

**Published:** 2022-11-03

**Authors:** Hollis C. Karoly, Mark A. Prince, Noah N. Emery, Emma E. Smith, Cianna J. Piercey, Bradley T. Conner

**Affiliations:** Department of Psychology, Colorado State University, Fort Collins, Colorado, United States of America; University of the West of Scotland, UNITED KINGDOM

## Abstract

Cannabis is commonly used among people who drink alcohol, yet evidence on acute effects of co-use is conflicting. Two important variables that may influence the effects of cannabis and alcohol are cannabinoid content (i.e., the ratio of cannabidiol [CBD] and 9-tetrahydrocannabinol [THC]) as well as the order of use (i.e., cannabis before alcohol vs. alcohol before cannabis). Research is mixed regarding the acute imapct of cannabis on alcohol consumption and intoxication, with some studies suggesting additive effects of alcohol and cannabis, and others demonstrating negligible effects of combining these substances. Further complicating this, high-THC-content cannabis concentrates are increasingly popular on the legal-market, but to our knowledge, no studies have explored concentrate and alcohol co-use. In addition to cannabinoid content, order of use may influence intoxication and other acute effects, but is also understudied. Co-use studies typically administer a fixed dose of alcohol before cannabis, and there is a lack of data on the acute effects of cannabis before alcohol. Thus, there is a need for experimental co-use studies exploring the impact of cannabinoid content (particularly of highly potent cannabis concentrates) and order effects on intoxication. This study uses a federally-compliant mobile laboratory procedure to explore the effects of co-administration of legal-market cannabis concentrates with a moderate alcohol dose (.8g/kg) in a sample of community participants who regularly use alcohol and cannabis. The study will also explore alcohol and cannabis order effects (cannabis before alcohol vs. alcohol before cannabis). Outcomes are objective intoxication (measured using blood cannabinoid level, heart rate, psychomotor performance and breath alcohol level [BrAC]) and subjective intoxication (assessed via self-report measures). Overall, this study may influence harm-reduction recommendations for individuals who drink alcohol and use cannabis.

## Introduction

Cannabis and alcohol are frequently used together, but there is conflicting evidence regarding the impact of cannabis on alcohol consumption and intoxication. Some studies suggest that cannabis reduces alcohol intake [1], others suggest that it increases alcohol intake [2], negative consequences [3] and intoxication [4, 5], and some studies show no effects of co-use on intoxication above the effects of single-substance use [6, 7].

Several individual-level variables may impact the alcohol-cannabis relationship, including understudied variables such as cannabinoid content (i.e., ratios of Δ9-tetrahydrocannabinol [THC] and cannabidiol [CBD]) [8, 9] and the order in which alcohol and cannabis are consumed. Further complicating our understanding of the interplay between alcohol and cannabis is the fact that cannabis products containing high amounts of THC are increasingly popular within the legal, recreational cannabis market. As an example, the average THC concentration in flower cannabis used in Colorado is 20% [10]. Widely-used, highly-potent cannabis concentrates contain up to 90% THC [11]. At present, we are unaware of any research that has explored the combined effects of alcohol and cannabis concentrates.

Due to the respective pharmacokinetics of cannabis and alcohol, the order in which they are used during a co-use event may influence intoxication. There is limited research on the effects of order of use on objective and subjective intoxication. The few existing alcohol and cannabis co-administration studies have administered fixed doses of alcohol prior to cannabis, and have shown that this order of use is associated with reduced peak blood alcohol content level (BAC), longer time to reach peak BAC, higher blood-THC levels, longer duration of intoxication and increased subjective intoxication and impairment [4, 12–17] compared to when alcohol or cannabis is used alone. One event-level, observational study found that using cannabis first on a given co-use day was associated with lower daily alcohol consumption, but greater daily cannabis consumption [18]. Besides this study, co-use research has largely ignored the effects of using cannabis before alcohol. However, colloquial wisdom states that using cannabis before alcohol is associated with fewer negative consequences, and the “cannabis first” method is thus a popular choice for people who co-use [18]. Further research is needed to understand the effects of order of use on intoxication, as well as to explore the impact of cannabis potency and cannabinoid content on intoxication.

These questions are challenging for researchers in the United States to explore due to federal restrictions on cannabis research. Cannabis is a Schedule I drug, and the only legal sources of cannabis for research is the NIDA supply program or DEA-approved growers. To our knowledge, neither of these options currently offer cannabis concentrates for research. Studies examining cannabis concentrates as they are commercially available and used in the legal market are needed to explore how high-potency cannabis impacts alcohol use and make recommendations for public health. This project will leverage a mobile laboratory—an innovative method compliant with federal law that facilitates *naturalistic*, *observational studies* of legal-market cannabis—to investigate these relationships. This method has been well-established by researchers in the state of Colorado [11, 19–23], where legal-market cannabis is prevalent and individuals have access to a wide variety of recreational cannabis products. Using this observational design, we will be able to explore the effects of legal-market cannabis concentrates as they are used in the real world.

This protocol describes an application of a novel research methodology, in which we are testing the effects of cannabis concentrates co-administered with a moderate dose of alcohol (.8g/kg) on objective intoxication (measured using blood cannabinoid level, heart rate, psychomotor impairment and breath alcohol level [BrAC, used as a proxy for BAC]) and subjective intoxication (assessed via self-report) in a sample of heavy-drinking community participants who regularly use cannabis. Our design also allows us to explore the potential role of order effects (alcohol before cannabis vs. cannabis before alcohol). The protocol utilizes a federally compliant mobile laboratory which is fully equipped to travel to participants’ places of residence in order to conduct the study. The use of a mobile laboratory to study legal market cannabis products is a well-established method in Colorado [11, 22, 24–26] and allows participants to use legal-market cannabis concentrates *ad libitum* inside their homes and undergo testing immediately in the mobile lab. This protocol allows us to investigate the effects of cannabis concentrates and alcohol on intoxication and impairment, and compare effects when cannabis is used immediately before alcohol and when cannabis is used immediately after alcohol. Specifically, participants are asked to consume as much or as little of their preferred cannabis concentrate product as they wish (following federal law and consistent with prior research on this topic leveraging the mobile laboratory [24–26]) and half of the participants are asked to consume the standard alcohol dose before using the cannabis concentrate (order AC), while the other half are asked to use their cannabis concentrate before consuming the standard alcohol dose (order CA).

### Study aims

#### Aim 1

Characterize the effects of cannabis concentrate and alcohol co-administration on intoxication and impairment outcomes over a period of 5-hours among both orders (CA and AC).

#### Aim 2

Compare intoxication and impairment levels in individuals who consume cannabis before alcohol (AC) and those who consume alcohol before cannabis (CA).

## Materials and methods

### Sample selection

Participants will be recruited from the community using online advertisements and flyers posted locally. Individuals interested in participating will complete screening procedures conducted by a trained research assistant. Inclusion criteria are as follows: 1) 21–65 years old, 2) Heavy drinkers, as defined by the National Institute on Alcohol Abuse and Alcoholism (NIAAA) criteria for heavy alcohol use (for males, consuming more than 4 drinks on any day or more than 14 drinks per week, and for females, consuming more than 3 drinks on any day or more than 7 drinks per week), 3) Use legal-market cannabis at least 3x/week in the past 3 months, 4) Have experience using cannabis concentrates within the last year, 5) Vaccinated and boosted against Covid-19, 6) Willing to wear a mask throughout the duration of their mobile lab appointment. Exclusion criteria are as follows: 1) Daily tobacco use, 2) Diagnosed with or seeking treatment for alcohol use disorder (AUD) or other substance use disorder (SUD), 3) Cannot be pregnant, breastfeeding or trying to become pregnant, 4) Meet criteria for psychotic, bipolar or major depressive disorder with suicidal ideation, or history of these disorders, 5) Current use of psychotropics (except anti-depressants) or steroids, 6) Report illicit drug use in the past 60-days or fail drug screen, 7) Report having a major medical condition contraindicating alcohol and/or cannabis consumption (e.g., liver disease, heart disease) or report being told by a doctor that it is unsafe to consume alcohol or cannabis due to a medical condition).

### Study design and procedures

This study has been approved by our University Institutional Review Board (IRB) and tests the effects of co-administering cannabis concentrates and alcohol on objective intoxication (measured using blood cannabinoid level, heart rate, psychomotor performance and breath alcohol level [BrAC, used as a proxy for BAC]) and subjective intoxication (assessed via self-report) in a sample of heavy drinking community participants who regularly use cannabis. During the study half of the participants in each product group consume the standard alcohol dose before using the cannabis concentrate (order AC) and the other half use the cannabis concentrate before the alcohol dose (order CA).

All participants in the study provide informed consent virtually and complete baseline surveys online. They then complete a 5-hour session in the mobile laboratory. Prior to the mobile lab session, participants are asked to obtain their preferred cannabis concentrate product from a legal-market dispensary, and retain the packaging so that we can collect data on the THC and CBD (if applicable) potency of the product for use as a covariate in analyses. Asking participants to use their preferred product as they normally would during the study increases the external validity of results.

**Fig 1:**
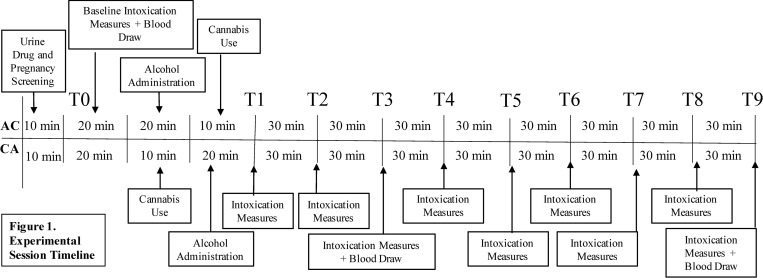
Experimental timeline. Detailed experimental session timeline for individuals completing the session in order AC (alcohol before cannabis) and CA (cannabis before alcohol). The “Intoxication Measures” completed at T0-T9 include BrAC, heart rate, subjective effects of alcohol, drug effects, measures of emotion and the Druid task.

At the start of the mobile laboratory session, research assistants check participant IDs to ensure that they are over 21 years of age. Participants then undergo a breathalyzer test, urine drug screen and pregancy test (if applicable). If breathalyzer readings are above 0.00, the appointment is rescheduled. If a participant is pregnant or tests positive for ilicit drugs besides cannabis, they are dropped from the study for safety reasons. Next, all participants complete a battery of subjective and objective intoxication measures (see “Outcomes”), take heart rate measurements and provide 10 mL of blood to assay blood-levels of THC, CBD and other minor cannabinoids. These procedures all take place within the mobile lab. Individuals completing the experiment following Order AC (alcohol before cannabis) undergo a standard alcohol administration procedure in which they ingest .8g/kg of alcohol (vodka mixed with orange juice) based on their body weight. In accordance with standard alcohol administration procedures [27–29], participants are asked to consume the alcohol within 20 minutes. Next, participants go inside their place of residence without the researchers and use their desired cannabis concentrate product ad libitum. They are asked to provide a picture of the product packaging (to confirm THC and CBD content) and weigh the product before and after use. While participants are inside their residence, they are connected to researchers via Zoom on their tablet or mobile device, following established procedures [30]. Researchers can thus observe participants using their cannabis product inside their residence via Zoom. The number of puffs, duration of inhalation, and the participants’ time spent holding their breath upon product inhalation are all recorded and such smoking topography information could all be covaried in subsequent analyses as appropriate. After using their concentrate, participants immediately return to the mobile lab and the research assistant measures their BrAC, heart rate and subjective and objective intoxication every 30 minutes for the next 4 hours. Participants remain in the mobile lab with the researchers for the remainder of the experiment. Peak BrAC is expected to occur 60 minutes after alcohol administration is complete [31], thus a second blood draw occurs 60-minutes after alcohol administration. A third blood draw occurs at the end of the experiment. Individuals in Order CA (cannabis before alcohol) complete the same procedures, but consume their cannabis concentrate (inside their residence) following the first intoxication battery and blood draw, and undergo alcohol administration immediately after using the cannabis. The rest of the experiment is identical for all participants, regardless of whether they are in Order AC or Order CA. Fig 1 shows a detailed session timeline.

The study is currently recruiting and enrolling participants and is planned to be run over a 3-year period. As this study has only recently gotten underway, this study protocol does not report on any existing data. Thus, the Data Availability policy is not applicable.

### Statistical power and sample size justification

The primary goal of this study (Aim 1) is to test the effect of alcohol and cannabis concentrate co-administration on subjective intoxication, objective intoxication, heart rate, blood-THC and BAC. Aim 1 will be tested with level 1 direct effects within a multilevel model. For aim 2, we plan to compare these effects across two administration orders: alcohol before cannabis and cannabis before alcohol. Thus, aim 2 is tested using cross-level interactions within a multilevel model. Each participant in this sample will provide 10 momentary assessments. We estimate that N = 60 participants is sufficient to power the study to detect small level 1 direct effects and medium-sized, cross-level interaction effects. We base our estimates on a recent simulation study [32] that examined power for 2-level multilevel models across a range of study conditions with level-2 units (e.g., number of participants) ranging from 30 to 200 and level-1 units (e.g., number of momentary assessments per participants) ranging from 3 to 30. These simulations explored level 1 direct effects, level 2 direct effects and cross-level interactions (level 2 variables predicting level 1 relations). They concluded that studies with samples in these ranges were adequately powered to examine direct level 1 and level 2 effects as well as cross-level interactions across a wide range of intraclass correlations (ICCs) and at least medium-sized random slope variances (RSV) for cross-level interactions. Importantly, the cross-level interactions required the largest number of participants to detect effects, thus powering our study for cross-level interactions provides the most conservative estimate of power and means that we are sufficiently powered for all level 1 and level 2 effects. One factor in determining power for cross level interactions is RSV. For a medium-sized RSV, we have adequate power to detect medium sized cross-level interactions with a total of 60 participants at 10 observations per person [32].

Note that we chose to power the study for medium effect sizes rather than small effect sizes as medium effects are more meaningful in terms of the clinical and public health implications of the present study. Specifically, identifying medium (or large) group differences in intoxication across product type will allow us to make recommendations to consumers about which products to use to minimize intoxication when co-using alcohol and cannabis. Identifying only small group differences in intoxication (that may or be not be noticeable to individual consumers using the products) would likely not be as helpful for informing such recommendations. Further, the present study describes an application of a highly novel research design, which could inform future pilot studies or large-scale trials. We will therefore be adequately powered for all direct effects at level 1 or level 2 regardless of ICCs and will be adequately powered for cross level interactions for RSVs that are at least medium sized.

### Primary outcomes

**Breath alcohol concentration (BrAC)** is measured throughout the experiment using a breathalyzer, as a commonly used proxy for BAC [33].

**Heart rate** serves as a highly reliable objective measure of acute cannabis effects[34] and is monitored using a basic fingertip pulse oximeter throughout the study session.

**Blood cannabinoid levels** for THC, CBD and two THC metabolites (THC-COOH and 11-OH-THC) are measured immediately after the cannabis use period, as well as at peak BAC (one-hour post-alcohol consumption) and at the end of the experiment (4 hours post-alcohol consumption).

The **Biphasic Alcohol Effects Scale (BAES)** [35], which measures stimulant and sedative effects of alcohol is administered before alcohol and cannabis use and every 30 minutes thereafter.

**The Subjective High Assessment Scale (SHAS)** [36], a standard self-report measure used to assess subjective responses to alcohol, is administered before alcohol and cannabis use and every 30 minutes thereafter.

**A visual analog scale (VAS) for rating cannabis intoxication** [37] is administered before alcohol and cannabis use and every 30 minutes thereafter.

The **Druid**^®^ **mobile application** is administered via tablet to assess cognitive and psychomotor motor impairment due to any cause or combination of causes including cannabis [21, 38], alcohol [39], illicit drugs, prescription medications, chronic medical conditions, illness, and fatigue. The app has users perform four tasks, presented in quick succession, that measure cognitive-motor performance by assessing balance, decision-making accuracy, reaction time, hand-eye coordination, and time estimation under conditions of divided attention. Taking only 3 minutes, Druid can be administered repeatedly in a single session to assess changes in impairment. Scores range from 0–100 and can be interpreted as follows: <44 = no impairment; 44–48 = mild impairment; 48–52 = moderate impairment; 52–57 = high impairment; 57–62 = very high impairment; and >62 = severe impairment. In this study, Druid is administered before alcohol and cannabis use and every 30 minutes thereafter.

### Secondary outcomes

**Positive and Negative Affect Schedule–Expanded Form (PANAS-X)** [40], a reliable measure of emotion, is administered before alcohol and cannabis use and every 30 minutes thereafter.

**Drug Effects Questionnaire (DEQ)** [41], a 5-item visual analog scale used to measure the strength of cannabis and its desirable effects, is administered before alcohol and cannabis use and every 30 minutes thereafter.

**Addiction Research Center Inventory** [42], which measures subjective effects of cannabis and drug-induced euphoria, stimulant-like effects, intellectual efficiency and energy, sedation, dysphoria, and other somatic effects, is administered before alcohol and cannabis use and every 30 minutes thereafter.

### Data analysis and statistical plan

To account for the effects of clustering resulting from the repeated data collection the best practice approach to testing study hypotheses is to use multilevel structural equation modeling (MSEM [43, 44]). MSEM represents advancement over traditional multi-level models, which are typically limited to univariate applications [45]. MSEM allows for multivariate structural equation models with nested data. Aim 1 focuses on examining the change in outcomes of interest over time and aim 2 compares these changes across groups (order AC vs. CA). For aim 1, we will examine changes over the 10 assessments on primary outcomes (e.g., subjective intoxication, psychomotor impairment, heart rate, blood-THC, BrAC) captured at baseline and every 30 minutes from ingestion to 4 hours post-use of alcohol and cannabis, at the momentary level. Change over time will be modeled by estimating a random slope representing the change in each indicator within each participant over the 5-hour study period. The aim 2 group comparisons will be examined using cross-level interactions [46]. Aim 2 will examine differences in changes over time across order conditions.

Study aims will be addressed using a generalized linear model framework (GLM) within multilevel structural equation models (MSEM) because substance use outcomes are often highly skewed and overdispersed count variables. Thus, we will use GLMs with a negative binomial specification to account for the distributions of the outcome variables [47]. Analyses will be run in Mplus Version 8.0, which easily accommodates missing data using full information maximum likelihood or multiple imputation, which is the best-practices approach to handling missing data in a multilevel framework [48].

### Ethical considerations

This protocol has approval from our University’s Institutional Review Board (Protocol Approval #2161). Primary ethical considerations for the study are related to acute alcohol and cannabis administration. Steps are taken to minimize risk to participants as a result of the acute effects of alcohol including those based on guidelines provided by the NIAAA Recommended Council Guidelines on Ethyl Alcohol Administration in Human Experimentation [49]. Participants are excluded if they have a medical condition that contraindicates alcohol consumption, are diagnosed with AUD or SUD, or are in treatment for, or seeking treatment for, AUD or SUD. Study staff closely monitor participant BrAC via breathalyzer throughout the experiment and participants are required to remain in the laboratory until their BrAC falls below .03 g/dL. The alcohol administration procedure is designed to bring BrAC to a maximum of .089, which is approximately at the legal limit. The amount of alcohol provided during the session should not exceed the dosage level consistent with participants’ typical drinking practices. Participants may also experience adverse effects resulting from consumption of cannabis concentrates. Concentrates confer rapid intoxication effects [50] and are more likely to produce anxiety, agitation, paranoia, and psychosis in comparison to other cannabis products [51]. However, because inclusion criteria for study participation require that individuals be regular (at least 3x/week) cannabis users and individuals are required to have experience using cannabis concentrates, it is unlikely that participants would experience cannabis-related negative side effects that differ from what they experience in their day-to-day life using cannabis. In addition, simultaneous alcohol and cannabis consumption may intensify the intoxication effects of both drugs [52].

When having blood drawn, participants may experience some discomfort as a result of the needle prick in the arm, as well as bruising or slight bleeding. Infection is unlikely, given that the needle is sterile and disposable. Participants may feel lightheaded or faint when blood is drawn, but the volume taken is small, and the mobile laboratory is stocked with snacks and water for participants should they become lightheaded. An individual trained in drawing blood is always present for the duration of the study session. Participants may also experience discomfort associated with filling out questionnaires (particularly regarding items that reference medical conditions, health- related behaviors, and stigmatized behaviors, such as drug use). They are forewarned of this possibility and notified that discomfort with questions may be handled by discussing the resultant discomfort with a trained clinician (e.g., the study PI or Co-Is, all of whom are licensed psychologists) or by declining to answer any questions. Participants are informed that they have the right to withdraw consent at any point throughout the duration of the study.

## Discussion

This study addresses questions regarding the impact of co-using cannabis concentrates alongside alcohol, with a particular focus on the order in which alcohol and cannabis are used. We acknowledge several notable limitations inherent in this observational design. Specifically, to adhere to federal law regarding research on legal market cannabis products, the researchers do not procure the cannabis products for participants and cannot administer the products to participants. Thus, the project does not necessitate FDA or DEA involvement or approvals. This also means we do not need to use NIDA-provided cannabis or receive a determination from the US Department of Health and Human Services stating that we are qualified to conduct the project. However, because individuals in the study are self-administering their own preferred cannabis during the study session, we are unable to strictly control the amount of cannabis consumed by participants in the study to ensure that all participants use the same amount. We will, however, collect data on the cannabinoid content of the products people use (i.e., having them submit a photo of the project packaging) and can include this information, as well as cannabinoid blood levels to control for THC exposure during the session. Further, researchers will be communicating with participants over Zoom an observing them using their cannabis, so we could control for numerous additional aspects of smoking topography as necessary. Because the goal of this work is to understand the effects of cannabis as it is used in the real world, we emphasized external validity over tight experimental control. A randomized controlled trial (RCT) would increase internal validity, but RCTs are not currently permitted for legal market cannabis. Future studies may choose to follow up on these results using more tightly controlled dosing or administration procedures following federal regulatory guidelines. Overall, given the limited research on co-administration of alcohol and legal-market cannabis concentrates, this highly novel research design may generate useful data despite the notable design limitations. Specifically, results from this project are expected to promote the advancement of the cannabis sciences and may have harm reduction implications for heavy-drinking individuals who use cannabis concentrates.
