# Correction: Protocol for a mobile laboratory study of co-administration of cannabis concentrates with a standard alcohol dose in humans

**DOI:** 10.1371/journal.pone.0322401

**Published:** 2025-04-08

**Authors:** Hollis C. Karoly, Mark A. Prince, Noah N. Emery, Emma E. Smith, Cianna J. Piercey, Carrie Cuttler, Bradley T. Conner

Carrie Cuttler should be included in the author byline. Carrie Cuttler should be listed as the sixth author, and his affiliation is 2: Department of Psychology, Washington State University. The contributions of this author are as follows: conceptualized ideas, developed or designed a methodology, contributed reagents/materials/analysis tools, provided activity planning and execution for the research.

The correct citation is: Karoly HC, Prince MA, Emery NN, Smith EE, Piercey CJ, Cuttler C (2022) Protocol for a mobile laboratory study of co-administration of cannabis concentrates with a standard alcohol dose in humans. PLoS ONE 17(11): e0277123. https://doi.org/10.1371/journal.pone.0277123
